# *Cryptococcus gattii* Infections in Multiple States Outside the US Pacific Northwest

**DOI:** 10.3201/eid1910.130441

**Published:** 2013-10

**Authors:** Julie R. Harris, Shawn R. Lockhart, Gail Sondermeyer, Duc J. Vugia, Matthew B. Crist, Melissa Tobin D’Angelo, Brenda Sellers, Carlos Franco-Paredes, Monear Makvandi, Chad Smelser, John Greene, Danielle Stanek, Kimberly Signs, Randall J. Nett, Tom Chiller, Benjamin J. Park

**Affiliations:** Centers for Disease Control and Prevention, Atlanta, Georgia, USA (J.R. Harris, S.R. Lockhart, R.J. Nett, T. Chiller, B.J. Park);; California Department of Public Health, Richmond, California, USA (G. Sondemeyer, D.J. Vugia);; Georgia Department of Public Health, Atlanta (M.B. Crist, M.T. D’Angelo); Phoebe Putney Memorial Hospital, Albany, Georgia, USA (B. Sellers, C. Franco-Paredes);; Hospital Infantil Federico Gomez, Mexico City, Mexico (C. Franco-Paredes);; New Mexico Department of Health, Santa Fe, New Mexico, USA (M. Mkvandi, C. Smelser);; Moffitt Cancer Center, Tampa, Florida, USA (J. Greene);; Florida Department of Health, Tallahassee, Florida, USA (D. Stanek);; Michigan Department of Community Health, Lansing, Michigan, USA (K. Signs);; Montana Department of Public Health and Human Services, Helena, Montana, USA (R.J. Nett)

**Keywords:** Cryptococcus, gattii, meningitis, pneumonia, fungal, fungi, Pacific Northwest, United States

## Abstract

Clonal VGII subtypes (outbreak strains) of *Cryptococcus gattii* have caused an outbreak in the US Pacific Northwest since 2004. Outbreak-associated infections occur equally in male and female patients (median age 56 years) and usually cause pulmonary disease in persons with underlying medical conditions. Since 2009, a total of 25 *C. gattii* infections, 23 (92%) caused by non–outbreak strain *C. gattii*, have been reported from 8 non–Pacific Northwest states. Sixteen (64%) patients were previously healthy, and 21 (84%) were male; median age was 43 years (range 15–83 years). Ten patients who provided information reported no past-year travel to areas where *C. gattii* is known to be endemic. Nineteen (76%) patients had central nervous system infections; 6 (24%) died. *C. gattii* infection in persons without exposure to known disease-endemic areas suggests possible endemicity in the United States outside the outbreak-affected region; these infections appear to differ in clinical and demographic characteristics from outbreak-associated *C. gattii*. Clinicians outside the outbreak-affected areas should be aware of locally acquired *C. gattii* infection and its varied signs and symptoms.

*Cryptococcus gattii*, a fungal pathogen found in the environment, is associated with soil and decaying organic debris. Infection in humans results from inhalation of spores from the environment and typically causes pneumonia or meningitis (*1*,*2*); the incubation period is thought to be 2–13 months, although it may be longer (*3*,*4*). Unlike the related species *C. neoformans*, which is distributed globally and is a common opportunistic infection in HIV-infected or severely immunocompromised persons, *C. gattii* typically affects patients without HIV infection (*1*,*2*,*5*–*8*), and its environmental distribution is thought to be more limited (*9*–*11*). *C. gattii* infection is typically considered more difficult to treat than *C. neoformans* infection and requires longer and more aggressive treatment (*1*,*7*,*8*,*12*,*13*).

Before 1999, clinical isolates of *C. gattii* were rare in North America; a small number of cases were reported, mostly in southern California and in Hawaii (*9*,*10*,*14*,*15*). However, since 2004, an outbreak of *C. gattii* cryptococcosis has been ongoing in British Columbia, Canada, and the US Pacific Northwest states of Washington and Oregon (*2*,*5*). Approximately 100 *C. gattii* cases have been reported from Washington and Oregon. The US Pacific Northwest outbreak is characterized by infection with 3 clonal *C. gattii* strains (VGIIa, VGIIb, and VGIIc), 2 of which are uncommon outside this region and 1 (VGIIc) that is unique to the region (*16*). Previously reported cases of *C. gattii* infection were in otherwise healthy patients who had severe central nervous system (CNS) disease (*1*). In contrast, most patients associated with the US Pacific Northwest outbreak have had respiratory symptoms and preexisting immunocompromising or other serious underlying medical conditions before becoming infected with *C. gattii* (*2*).

The outbreak in the US Pacific Northwest has increased interest in *C. gattii* among public health authorities and the US health community and resulted in efforts by the Centers for Disease Control and Prevention (CDC; Atlanta, GA, USA) to collect surveillance data on *C. gattii* infections from states outside that region. Whether these reported cases are an effect of the US Pacific Northwest outbreak on other areas of the United States and the implications for clinical care and broader surveillance have not been addressed. We summarize *C. gattii* cases reported to CDC from non–US Pacific Northwest states and discuss their implications.

## Methods

A case was defined as a culture-confirmed *C. gattii* infection in a resident of the United States outside of Oregon or Washington who had no known recent travel history (within 1 year) to these states and had illness onset after January 1, 2009. Cases were reported to CDC by state health departments or physicians who were treating patients with suspected *C. gattii* infection (typically because the patient was not infected with HIV). Isolates were sent to CDC for confirmation; isolates were plated on differential canavanine-glycine-bromothymol blue medium (*17*), and molecular type and subtype were identified by using multilocus sequence typing at 7 loci (*18*). Neighbor-joining trees were developed by using the multilocus sequence typing results and MEGA 4.0 software (www.megasoftware.net). State and local health department staff and treating physicians completed standardized case report forms with demographic and clinical information on case-patients and submitted these reports to CDC. Sites of infection were determined as follows: for pulmonary, blood, cerebrospinal fluid (CSF), or tissue specimens, culture was considered evidence of infection at that body site. Histopathologic examination results demonstrating *Cryptococcus* yeasts in tissue also was considered evidence of infection at the tissue site. Cryptococcal antigen in the CSF was considered evidence of CNS infection; however, serum cryptococcal antigen in the absence of a bloodstream cryptococcal isolate was not considered evidence of a bloodstream infection.

## Results

Twenty-five cases for which descriptive data were available were identified in Alabama (1 case), California (13), Florida (1), Georgia (5), Hawaii (1), Michigan (1), Montana (1), and New Mexico (2). Some cases have been published as individual case reports (*2*,*19*–*21*). Twenty-one (84%) patients were male; median age was 43 years (range 15–83 years) (Table 1, Appendix). All patients had illness onset during January 2009–May 2012.

**Table 1:** Clinical characteristics of patients with *Cryptococcus gattii* infection outside the US Pacific Northwest, 2010–2012*

Characteristic, n = 25†	No. (%)‡
Demographic	
Male sex	21 (84)
Sign or symptom	
Headache	16 (67)
Blurred vision§	8 (62)
Nausea	11 (46)
Fatigue	11 (46)
Weight loss	10 (42)
Vomiting	10 (42)
Cough	8 (33)
Fever	7 (29)
Loss of appetite§	3 (25)
Neck stiffness	5 (20)
Chills	4 (17)
Dyspnea	4 (17)
Night sweats	3 (13)
Photophobia	3 (13)
Chest pain§	1 (8)
Papilledema	2 (8)
Muscle pain	2 (8)
Seizure	1 (4)
Underlying conditions (*C. gattii* molecular type)	
None	16 (64)
Immunocompromising	5 (20)
Pulmonary sarcoidosis (VGI)	1
Diabetes, liver transplant (VGIIb)#	1
Unspecified immunocompromising condition (VGIII)#	1
Congenital hyper-IgE, i.e., Job syndrome (VGI)	1
Active lung cancer, receiving chemotherapy (VGIII)	1
Other underlying condition	4 (16)
History of prostate cancer, not on treatment (VGIII)	1
Diabetes, COPD, aortic stenosis (VGI)	1
Diabetes, RHD, restrictive lung disease, history of skin cancer (not on treatment) (VGI)#	1
Diabetes (VGIII)	1
Cryptococcomas (among those with images)§	
Lung, n = 23	14 (61)
Brain, n = 20	10 (50)
Lung and brain, n = 18	5 (28)
Sites with evidence of infection	
Any CNS site (CSF or brain)	19 (76)
Any pulmonary site (lung or sputa)	9 (36)
CNS only	12 (48)
CNS and pulmonary only	3 (12)
Pulmonary only	5 (20)
Blood only	1 (4)
CNS and blood only	2 (8)
CNS, blood, and pulmonary	1 (4)
CNS and leg tissue	1 (4)
Outcome	
Hospitalized	23 (92)
ICU admission‡	10 (48)
Died of *C. gattii* infection	6 (24)

*COPD, chronic obstructive pulmonary disease; RHD, rheumatic heart disease; CNS, centeral nervous system; CSF, cerebrospinal fluid; ICU, intensive care unit. †Patients’ median age was 43 years (range 15–83 years). ‡Proportions represent patients with available data. Complete data were not available for all patients. §Data not available for all patients. #Died of their cryptococcal infections.

All patients except 1 were symptomatic. The single case in an asymptomatic patient was diagnosed by a chest radiograph during a post-trauma hospitalization; the radiograph showed upper lobe abnormalities. Four patients died before diagnosis; among the 21 patients surviving to diagnosis, the median time from symptom onset to diagnosis was 32 days (range 2–263 days). The most common symptoms were headache (67%), blurred vision (62%), and nausea (46%) (Table 1, Appendix). Leukocyte counts for 17 patients at diagnosis ranged from 6,400–20,700/mm^3^ (median 11,700); CD4 counts, available for 4 patients, ranged from 92 cells/mm^3^ to 838 cells/mm^3^ (median 595 cells/mm^3^). Twenty-three (92%) patients were hospitalized for a median of 23 days (range 1–88 days); 10 (48%) of 21 patients with data required admission to the intensive care unit. Results of lung imaging were abnormal for 19 (83%) of 23 patients with images; 14 (61%) had documented lung cryptococcomas. Results of head imaging were abnormal in 15 (75%) of 20 patients with images; 10 (50%) patients had documented brain cryptococcomas. Nineteen (76%) patients had culture, histopathologic, or serologic evidence of CNS infection, either alone or with pulmonary, blood, or tissue infections (Table 1, Appendix). Nine (36%) patients had culture or histopathologic evidence of pulmonary infection; 5 had isolated pulmonary infections. For 4 patients, *C. gattii* was isolated from blood samples.

Sixteen (64%) patients were otherwise healthy at diagnosis (Table 1, Appendix). Five (20%) patients had immunocompromising conditions (Table 1, Appendix): pulmonary sarcoidosis (CD4 count of 92 cells/mm^3^), diabetes and liver transplant, an unspecified immunocompromising condition, active lung cancer (patient receiving chemotherapy), and congenital hyper-IgE syndrome (Job syndrome). Four (16%) additional patients had nonimmunocompromising underlying disease at diagnosis. For the 12 patients with documented HIV test results, all results were negative.

Six patients (ages 18, 36, 39, 56, 68, and 82 years) died, all from their *C. gattii* infections (Table 2, Appendix). Three were previously healthy, 1 had multiple nonimmunocompromising underlying medical conditions, and 2 were immunocompromised. One of the 3 previously healthy patients (18-year-old woman) had a fulminant course of illness that led to death 2 weeks after onset. This patient sought treatment at a community hospital with headache, fever, and flat affect; she received ceftriaxone for a urinary tract infection and was hospitalized for suspected pseudoseizures. On day 4 of her hospitalization, she became lethargic and had a low-grade fever (100.8°F). A lumbar puncture (opening pressure >60 mm H_2_O) showed evidence of yeast; she was given liposomal amphotericin B and 5-flucytosine and was transferred to a referral hospital, where she received a lumbar puncture to drain CSF and endotracheal intubation for mechanical ventilation in the intensive care unit. Despite intensive supportive care, she died 2 days later of brain stem herniation caused by persistent elevated intracranial pressure (ICP).

**Table 2:** Characteristics of patients who died of *Cryptococcus gattii* infections acquired outside the Pacific Northwest, US, 2010–2012*

Age, y/sex	State	Onset to diagnosis, d	Diagnosis to death, d/cause of death	Underlying conditions	Initial presentation/ clinical evaluation	Leukocyte count	CD4, cells/mm^3^	Sites yielding evidence of infection	Cg type	Shunt to manage elevated intracranial pressure?	Imaging results	Initial antifungal treatment
18/F	GA	10	4/cryptococcal infection (herniated brain stem)	None	HA, BV, WL, fever, photophobia, loss of appetite, fatigue, hearing loss	ND	ND	CNS only	VGIII	Yes	No head imaging results; pneumonia	Ambisome, 5-flucytosine
36/M	CA	7	0/cryptococcal infection	Unspecified immunocompromising condition	HA, NS, cough, dyspnea, fatigue, AMS	ND	ND	CNS and blood	VGIII	NA	Pneumonia (thought to be bacterial); no head imaging	None
39/M	GA	2	263/shunt blockage	None	HA, BV, chills, papilledema	15,500	217	CNS only	VGI	Yes	Head imaging showed multiple Virchow-Robin spaces, basal ganglia cortex cryptococcoma; pneumonia	Ambisome, 5-flucytosine; corticosteroid at initial discharge
56/M	NM	113	Died before diagnosis/cryptococcal infection	None	HA, NS, BV, WL, fatigue, muscle pain, AMS, confusion	12,600	560	CNS, lung, blood	VGIII	NA	Normal head imaging; pleural effusion	None
68/F	CA	41	Died before diagnosis/cryptococcal infection	Liver transplant, dialysis-dependent ESRD, diabetes. Multiple recent hospitalizations for VRE of leg, recurrent pleural effusion.	Nausea, fatigue, confusion	20,700	ND	Blood only	VGIIb	NA	Abnormal chest imaging (no further information); normal head imaging	None
82/F	CA	263	Died before diagnosis/cryptococcal infection	H/o squamous cell carcinoma (not on treatment), CHF, RHD, endocarditis, restrictive lung disease, diabetes. *Pseudomonas aeruginosa* infection.	Fatigue, WL	15,900	ND	Lung only	VGI	NA	Pneumonia, right upper lung cryptococcoma	None

*Cg,, Crylptococcus gattii ; GA, Georgia; HA, headache; BV, blurred vision; WL, weight loss; ND, no data. CNS, central nervous system; CA, California; NS, neck stiffness; NM, New Mexico; AMS, altered mental status; NA, not applicable; ESRD, end-stage renal disease; VRE: vancomycin-resistant *Enterococcus* infection; CHF, congestive heart failure. RHD: rheumatic heart disease.

In addition to this patient, 4 patients died before (or on the day of) their diagnoses, and 1 died 263 days after diagnosis (from shunt blockage). Three of the 4 who died before diagnosis had bloodstream infections. Three of the 4 female patients died; female sex (relative risk [RR] 5.3, 95% CI 1.6–17.3; p = 0.03) and having cryptococci isolated from blood (RR 5.3, 95% CI 1.6–17.3; p = 0.03) were significantly associated with increased risk for death. Death was not associated with other clinical or demographic variables.

Of the 21 patients surviving to diagnosis, treatment data were available for 19 (90%). All 17 who survived to diagnosis with bloodstream or CNS infections were initially given amphotericin B or liposomal amphotericin B; 16 also were given 5-flucytosine or fluconazole, and 1 was given amphotericin B alone. Of the 4 with isolated pulmonary infections who survived to diagnosis, 1 was given fluconazole alone, and 1 received amphotericin B and flucytosine; for 2, no treatment data were available.

Seven patients were given corticosteroids to treat their infection. One patient was given interferon. Twelve (57%) of 21 patients surviving to diagnosis had diagnostic or interventional surgeries, including 6 who needed a ventriculoperitoneal shunt or lumbar drain for refractory elevated ICP, 4 who needed brain or lung biopsies, 1 requiring placement of a chest tube for pleural effusion, and 1 requiring leg surgery at the infection site (*21*). Hydrocephalus developed in 4 (22%) of 18 patients with data, and cranial nerve palsies developed in 5 (38%) of 13 with data.

*C. gattii* isolates from patients were typed as VGI (11 [44%]), VGIII (11 [44%]), VGII (non–outbreak strain; 1 [4%]), and VGIIb (2 [8%]) (Table 3). Fifteen sequence types were represented among the 25 isolates, indicating a high degree of genetic diversity (Figure). One of the 2 VGIIb isolates was from an immunocompromised female patient from California with unknown travel history; the other was from an immunocompetent man from Florida with a leg infection and meningitis who reported no out-of-state travel for the past 20 years (*21*) (Table 1, Appendix; Table 3). Of the 13 California patients (reported from northern and southern California), 9 had unknown travel history, 2 had traveled to Mexico, and 2 had traveled to a central or eastern state in the year before their illnesses (Table 3). Ten patients (excluding the California patients) reported no exposure to known disease-endemic areas for at least the past year; 7 patients (1 from Alabama, 3 from Georgia, the Florida patient, the Montana patient, and 1 of the 2 New Mexico patients) reported no out-of-state travel for at least the past year (Table 3); 2 of these patients (New Mexico and Montana) were incarcerated, both for <1 year, in their home states before their illness onset. Eight had outdoor exposures involving construction or gardening during the year before their illness.

**Table 3:** Genotype of *Cryptococcus gattii* infection and known travel history of infected patients from outside the US Pacific Northwest, 2009–2013

State	Genotype	Known travel history
Alabama	VGI	No travel for many years; decades before, lived in Hawaii, Australia, and Asia
California	VGI	Unknown
California	VGI	Past-year travel to St. Louis, Missouri
California	VGI	Unknown
California	VGI	Unknown
California	VGI	Past-year travel to Betheseda, Maryland
California	VGIII	Past-year travel to Mexico
California	VGIII	Unknown
California	VGIII	Unknown
California	VGIII	Unknown
California	VGIII	Unknown
California	VGIII	Past-year travel to Mexico
California	VGIII	Unknown
California	VGIIb	Unknown
Florida	VGIIb	No travel outside of Florida for 20 years (*21*)
Georgia	VGI	No past-year travel
Georgia	VGI	No past-year travel
Georgia	VGI	Unknown
Georgia	VGIII	No travel for at least 2 years
Georgia	VGIII	Past-year travel to North Dakota; travel 5 years before illness to Montana
Hawaii	VGII (not a/b/c)	Past-year travel to Grand Canyon, Arizona, and Las Vegas, Nevada
Michigan	VGIII	Past-year travel to Albuquerque, New Mexico, and Denver, Colorado
Montana	VGI	Florida travel 3 years before illness; incarcerated in Montana for 7 months before illness onset
New Mexico	VGI	Frequent travel to Mexico, including during year before illness
New Mexico	VGIII	Never outside of New Mexico; incarcerated in New Mexico for 2 months before illness (*20*)

**Figure:**
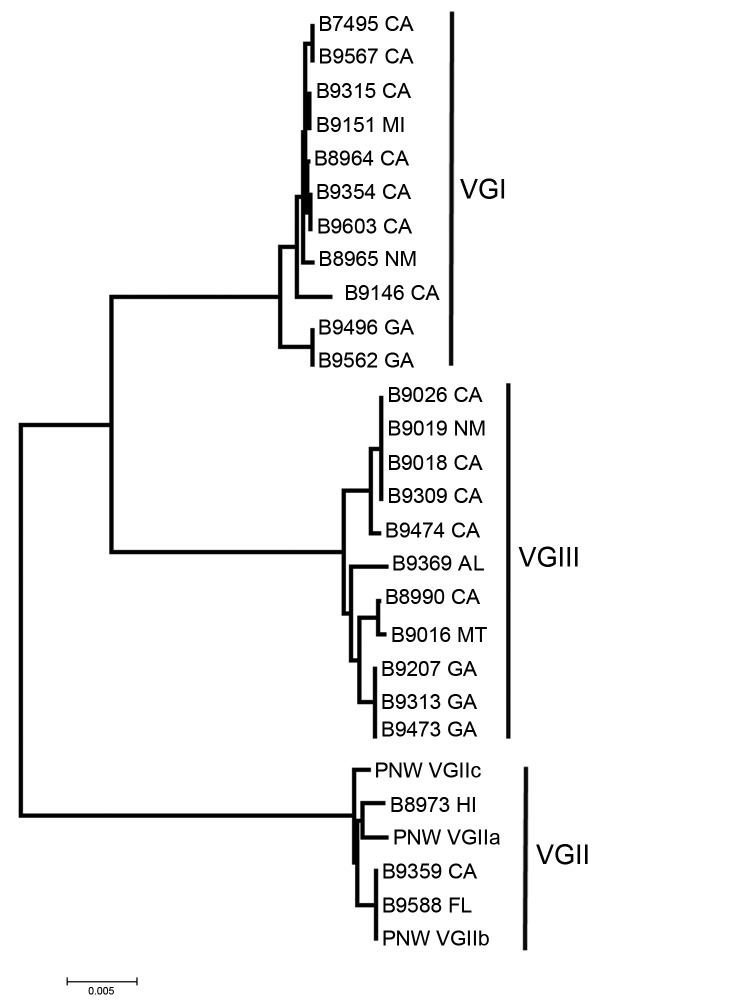
Neighbor-joining tree of US *Cryptococcus gattii* isolates from outside the US Pacific Northwest states of Washington and Oregon, 2009–2013. The tree was constructed by using multilocus sequence typing data from 7 unlinked loci. US Pacific Northwest *C. gattii* strains VGIIa, VGIIb, and VGIIc were added for reference.

## Discussion

We describe severe *C. gattii* infections in the United States, many probably acquired in states outside the outbreak-affected Pacific Northwest. Most of these cases differed clinically and genetically from outbreak strains of *C. gattii* that are causing disease in the US Pacific Northwest, and they are unlikely to be associated with the outbreak. Ten patients with travel history had not traveled to areas where *C. gattii* is known to be endemic for at least 1 year before illness onset, suggesting that *C. gattii* may be endemic in some areas of the United States outside the Pacific Northwest, and infections might be going unrecognized in these areas. California is increasingly recognized as an area where *C. gattii* is endemic, and the 13 patients from California could have acquired their infections locally or, perhaps in the case of the California and New Mexico patients with travel to Mexico, in Mexico. The high case-fatality rate indicates a serious public health issue, the scope of which is unknown.

Most patients in this report were otherwise healthy men infected with *C. gattii* molecular types VGI or VGIII who had CNS disease and positive CSF cultures; fewer patients had positive pulmonary cultures or respiratory symptoms. Many patients had visual disturbances, cranial nerve palsies, and hydrocephalus when seeking treatment or that developed during treatment. Several patients required surgical interventions to mediate persistent elevated ICP. Several patients were treated with corticosteroids, possibly to manage their elevated ICP; clinical treatment guidelines for cryptococcosis recommend judicious use of therapeutic corticosteroids under specific circumstances (*13*). Cryptococcomas in the lungs and brain were common among patients in this report; these masses are more common among patients with *C. gattii* than *C. neoformans* infection (*1*). These clinical and demographic characteristics, including the disproportionately high number of infected male patients, are similar to those reported for *C. gattii* infections from other *C. gattii*–endemic areas of the world, such as Australia and Papua New Guinea, where infections are caused primarily by molecular type VGI (*1*,*2*,*5*–*8*,*22*). Most patients in our report received appropriate initial antifungal treatment; of the 2 who died after diagnosis and initiation of treatment, 1 who died 4 days after diagnosis might have received the diagnosis too late for treatment to be effective, and the other died of infection-related causes but not directly from infection. However, 4 patients died before or at diagnosis (and thus before treatment could begin). Three of these 4 had prolonged illness before diagnoses, indicating a likely window of opportunity for earlier diagnosis and treatment that might have led to improved outcomes. Information clearly is needed to determine where in the United States *C. gattii* infections pose a public health concern and where clinicians need to maintain an elevated index of suspicion for *C. gattii* infection, particularly for otherwise healthy persons who have signs and symptoms of CNS disease, in whom cryptococcal infection might not be considered in the differential diagnoses.

The clinical signs and symptoms, host characteristics, clinical course, and molecular types of *C. gattii* infections reported here differ somewhat from those in the US Pacific Northwest outbreak. Most patients with *C. gattii* in the Pacific Northwest outbreak have underlying, often immunocompromising, medical conditions; are infected with *C. gattii* molecular types VGIIa, VGIIb, or VGIIc; and have pulmonary symptoms and positive pulmonary cultures (*2*). Approximately half of the Pacific Northwest patients are female and are a median of 56 years of age (2–13 years older than the median age of patients described here. More than half of patients in the Pacific Northwest outbreak were reported to be taking systemic oral steroids at diagnosis (*2*), a characteristic not identified among any patients in this report, and few of the patients in that outbreak reported blurred vision or required surgical interventions to mediate persistent elevated ICP during their infections (*2*).

The reasons for the clinical and demographic differences between outbreak-associated (VGIIa, VGIIb, and VGIIc) and non–outbreak-associated (other molecular types) *C. gattii* are not clear but may be related to case ascertainment differences, to the molecular type of *C. gattii* causing infection, to host differences, or a combination of these factors. Some studies have found that a patient’s immune status is critical to the clinical course of cryptococcal infection (*1*,*7*,*23*); for example, HIV-uninfected persons with *C. neoformans* infections in Asia have more visual and neurologic signs and symptoms consistent with uncontrolled elevated ICP than do HIV-infected patients (*23*,*24*). Inflammation can be a defining characteristic of cryptococcal infection in immunocompetent persons (*25*–*27*) and might increase the severity of certain symptoms (*25*,*28*,*29*), which accounts for differences in clinical signs and symptoms between the largely immunosuppressed US Pacific Northwest outbreak population and the mostly healthy population reported here. Perhaps the absence (or suppression) of an intact immune response among many *C. gattii* patients in the Pacific Northwest outbreak increases risk for cryptococcal infection and attenuates its clinical severity by reducing the inflammatory response, thus reducing outcomes such as hydrocephalus and persistent elevated ICP. In contrast, an intact immune response might perversely increase the severity of certain disease symptoms among otherwise healthy *C. gattii* patients, many of whom reside outside the Pacific Northwest. However, the different strains probably also preferentially infect different patient types, perhaps because of innate requirements of different *C. gattii* strains. Day et al. and Varma et al. also found that different genetic types of *C. neoformans* infected immunocompetent and immunocompromised (HIV-infected) persons living in comparable environments and circumstances (*30*,*31*); different *C. gattii* strains might also have predilections for different types of patients. Given the apparent correlation observed in the United States between molecular type of reported *C. gattii* infections and patient immune status, disentangling these characteristics might be difficult. All of the above factors—the site of infection, the strain, and the host—might be interdependent.

Our report has limitations. First, case reporting was carried out only in areas where laboratory staff, clinicians, or state health departments were aware of and interested in participating in CDC *C. gattii* surveillance. Thus, the distribution of reported *C. gattii* isolates might not represent the national distribution of *C. gattii* isolates outside the Pacific Northwest. Second, most infections in this report came from physicians specifically interested in obtaining species information for cryptococcal infections in their patients, usually because they were HIV uninfected; thus, we cannot make inferences about the frequency of *C. gattii* infections in HIV-infected persons nor the clinical course of *C. gattii* disease in HIV-infected persons from this report. Case ascertainment among patients in this group was also likely to differ from ascertainment in the Pacific Northwest, and comparisons between patients in each group should be interpreted with care. Additionally, co-existing pulmonary disease among these patients might be underreported. Although clinicians are likely to try to rule out CNS infection in patients with pulmonary cryptococcosis, such as those in the Pacific Northwest, patients with CNS cryptococcosis might be less likely to have pulmonary sites cultured because identifying the site will not influence treatment. Finally, we might have underestimated travel-associated infections; although most *C. gattii* infections are considered to have an incubation of 2–13 months (*3*,*4*), incubations as long as 3 years have been reported (*32*). Because most *C. gattii* incubation period data are based on persons exposed in the Pacific Northwest outbreak, and the outbreak is still relatively nascent, longer incubation periods might manifest as the time since exposure to the outbreak-affected region lengthens.

Although this case series might represent an emerging public health issue in states outside the US Pacific Northwest, it also might represent previously unrecognized disease in these states. Individual clinicians submitted multiple cases for this report, which suggests hotspots of environmental colonization, hotspots of individual interest, or both. However, *C. gattii* infections have been documented, albeit rarely, in at least some of these areas of the United States: in addition to the few cases identified in southern California, where *C. gattii* previously has been thought to be endemic, and Hawaii (*9*,*10*,*14*,*15*,*33*), a small number of cases also were reported in Georgia (*19*,*33*,*34*). Unlike the clonal outbreak strains found in the Pacific Northwest, the diversity and lack of clonality among the VGI and VGIII isolates from patients reported here indicates that these pathogens most likely have been propagating and diversifying in the United States for a long time. Anecdotal information about and treatment guidelines for *C. gattii*, primarily based on cases from Australia and Papua New Guinea, suggest that *C. gattii* infections might require more aggressive or longer treatment than *C. neoformans* infections (*1*,*7*,*8*,*12*,*13*).

An increased index of suspicion for *C. gattii* infection may be warranted in some areas of the United States; recognition of species *gattii* infection and early diagnosis might improve outcomes. Species identification of cryptococcal isolates requires specialized agar; however, most clinical laboratories do not identify isolates to the species level. Routine surveillance and species and molecular type identification for *C. gattii* outside the Pacific Northwest would enable identification of the true range of infections, and shed light on where diagnostics for *C. gattii* would be most useful.
